# Fatigue Properties of Hot-Dip Galvanized AISI 1020 Normalized Steel in Tension–Compression and Tension–Tension Loading

**DOI:** 10.3390/ma14237480

**Published:** 2021-12-06

**Authors:** Shatumbu Thomas Alweendo, Motoaki Morita, Kayo Hasegawa, Shinichi Motoda

**Affiliations:** 1 Graduate School of Marine Science and Technology, Tokyo University of Marine Science and Technology, Tokyo 135-8533, Japan; 2 Faculty of Marine Science and Technology, Tokyo University of Marine Science and Technology, Tokyo 135-8533, Japan; morita@kaiyodai.ac.jp (M.M.); neco_jkvv@yahoo.co.jp (K.H.); motoda@kaiyodai.ac.jp (S.M.)

**Keywords:** fatigue strength, stress ratio, hot-dip galvanized steel, normalized steel, fracture surface

## Abstract

Since hot-dip galvanizing causes a heat effect on cold-worked steel substrate and produces a coating layer comprised of distinct phases with varying mechanical properties, the fatigue mechanism of hot-dip galvanized steel is very complex and hard to clarify. In this study, AISI 1020 steel that has been normalized to minimize susceptibility to the heat effect was used to clarify the effect of the galvanizing layer on the tensile and fatigue properties. The galvanizing layer causes a reduction in the yield point, tensile strength, and fatigue strength. The reduction in the fatigue strength was more significant in the high cycle fatigue at *R* = 0.5 and 0.01 and in the low cycle fatigue at *R* = 0.5. The galvanizing layer seems to have very little effect on the fatigue strength at *R* = −1.0 in the low and high cycle fatigue. Since the fatigue strengths at *R* = 0.01 and −1.0 in the low cycle fatigue were strongly related to the tensile strength of the substrate, the cracking of galvanized steel was different than that of non-galvanized steel. The fatigue strength of galvanized steel at *R* = 0.5 dropped remarkably in the low cycle fatigue in comparison to the non-galvanized steel, and many cracks clearly occurred in the galvanizing layer. The galvanizing layer reduced the fatigue strength only under tension–tension loading. We believe that the findings in this study will be useful in the fatigue design of hot-dip galvanized steel.

## 1. Introduction

Although hot-dip galvanized steel structures are gaining popularity in various industries, there are still some reservations about the use of hot-dip galvanized steel in some applications such as structural steel members in bridges due to limited knowledge of their fatigue behavior and design recommendations [1,2,3]. Hot-dip galvanizing produces a coating layer comprised of different intermetallic phases of distinct mechanical properties [4,5]. The microstructure of the plating layer from a conventional zinc bath is comprised of a thin gamma (Γ) phase, which forms an interphase with the steel substrate, followed by the delta (δ) phase, then the zeta phase (ζ) phase, and the eta phase (η) made of pure zinc is at the outermost surface [6]. Other possible microstructures are dependent on the alloying additions to the zinc bath that can affect thermodynamic equilibria and the diffusion kinetics during the solidifying process [7,8,9,10,11]. Therefore, due to the many possible microstructures of the plating layer with the resulting mechanical properties and the requirement to preserve the mechanical performance of steel components after post-forming surface treatments such as galvanizing, the effect of the galvanizing layer on the fatigue strength of various steel types has been of much interest. Sirin [12] reported the formation of pre-cracks in the delta phase during solidification after hot-dip galvanizing and a reduction in fatigue strength. Wood et al. [13] reported a reduction of fatigue strength by the galvanizing layer when the layer de-bonded from the steel substrate due to the axial compressive stresses leading to significant stress concentration and crack initiation. Even in areas where de-bonding did not occur, significant cracking of the galvanizing layer was observed, and cracks propagated from the galvanizing layer to the steel substrate. Oechsner et al. [14] reported a 15% reduction in fatigue strength after hot-dip galvanizing and suggested a review of the available standards to incorporate guidelines on the fatigue design of hot-dip galvanized steel. Michailidis et al. [15] reported an effect of the galvanizing layer on the fracture mechanisms. Several researchers reported a heat effect on cold-worked and TRIP steel substrates in addition to a reduction of fatigue strength after hot-dip galvanizing [16,17,18,19,20,21]. Since hot-dip galvanizing causes a heat effect on the substrate and produces a coating layer with distinct phases of varying mechanical properties, the fatigue mechanisms of hot-dip galvanized steel become complex and hard to clarify. Therefore, there is a need for more research to clarify the effect of the galvanizing layer on the fatigue strength of steel.

To understand the effect of the galvanizing layer, it was necessary to use normalized steel because of its lower susceptibility to the heat effect on the dislocation structure and to investigate the effect of the galvanizing layer on tensile and fatigue strength in the low and high cycle fatigue. In this study, we clarified the effect of the galvanizing layer on the fatigue strength and fracture mechanisms of normalized AISI 1020 steel under different loading conditions (tension–compression and tension–tension). Since the steel is often applied to structures without investigating its properties after galvanizing, we pointed out the shortcomings of the applications. We believe that this study will contribute to the safe design of hot-dip galvanized steel structures.

## 2. Materials and Methods

### 2.1. Materials

**Figure 1:**
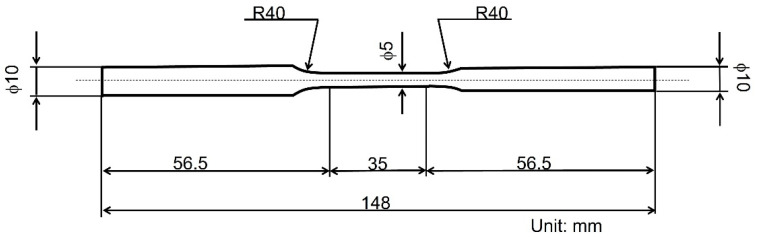
Schematic diagram of the tensile and fatigue test specimens.

The chemical composition of the commercial AISI 1020 steel used is presented in Table 1. A dog-bone shaped specimen with a gauge length of 35 mm and a diameter of 5 mm was used (Figure 1). The specimen was specifically designed for hot-dip galvanizing to allow for a much uniform plating layer, especially at curved sections of the specimen. The allowance of the Φ5 area is +0.1 and 0. The surface roughness of the Φ5 and R40 areas are <1.6 µm in arithmetic average roughness, which meets the JIS standards. As-received AISI 1020 steel specimens were normalized by heating in a muffle furnace (FO610, Yamato, Tokyo, Japan) to the austenitizing temperature region (870 °C), which was soaked for 60 min to allow for a full transformation, and cooling in ambient air. This specimen is referred to as non-galvanized steel in this study. The process parameters were decided based on the detailed description of the heat treatment of steel by Connor et al. [22]. The non-galvanized steel specimens were hot-dip galvanized at 450 ± 5 °C for about 3 min and cooled in water. This specimen is referred to as galvanized steel in this study.

### 2.2. Methods

Microstructures of non-galvanized and galvanized steel specimens were observed by optical microscopy (Eclipse LV150, Nikon, Tokyo, Japan), and the grain sizes were compared. The hardness values of the specimens were obtained by a micro-Vickers hardness testing machine (HM-200, Mitutoyo, Kawasaki, Japan) using a load of 0.02 kgf for 10 s. The specimens were tested for mechanical properties under static loading using a tensile testing machine (Shimadzu Autograph no. 79641, Shimadzu, Kyoto, Japan) at an initial strain rate of 4.76 × 10^−4^ s^−1^ and a crosshead speed of 1 mm/min. Fatigue testing experiments were carried out using a laboratory fatigue testing machine (Servopulser EHF-EUB5, Shimadzu, Kyoto, Japan) with a sine wave cyclic loading at a frequency of 10 Hz and stress ratios, *R*, ($\sigma_{min}$/$\sigma_{max}$) of −1.0, 0.01, and 0.5. The experiment was deliberately terminated when the specimen reached 10^7^ cycles without failure. The microstructure of the galvanizing layer and the morphologies of the fracture surfaces were examined using a scanning electron microscope (S-3500N, Hitachi, Tokyo, Japan) coupled with an energy-dispersive X-ray spectroscope (Octane pro, EDAX, Mahwah, NJ, USA). The crack initiation sites were identified on the fracture surfaces by observing the ratchet marks, ridges, and morphologies of the crack propagation area (stage II region).

## 3. Results

### 3.1. Microstructural Analysis

**Figure 2:**
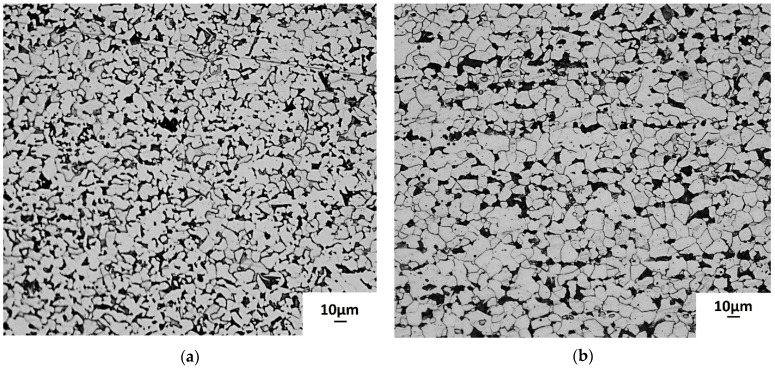
Optical micrographs for normalized AISI 1020 steel (**a**) before and (**b**) after hot-dip galvanizing.

**Figure 3:**
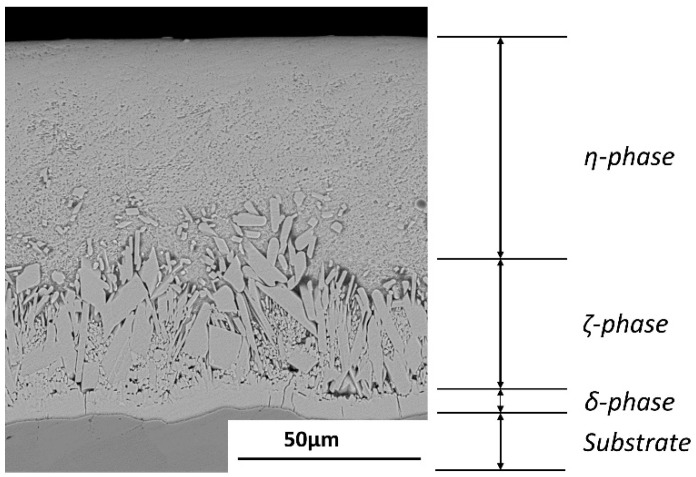
SEM (BSE) image of the different phases of the galvanizing layer.

The microstructures of non-galvanized steel and the substrate of galvanized steel substrates reveal equiaxed grains as shown in Figure 2. After galvanizing, there appears to be a slight grain growth caused by exposure of the substrate to the elevated zinc bath temperature as confirmed by the calculation of grain sizes presented in Table 2. Figure 3 shows the microstructure of the galvanizing layer, which reveals three distinct phases which were confirmed by optical observation and EDS analysis as the eta, zeta, and delta phases with a total thickness of 100.5 µm.

### 3.2. Mechanical Properties

**Figure 4:**
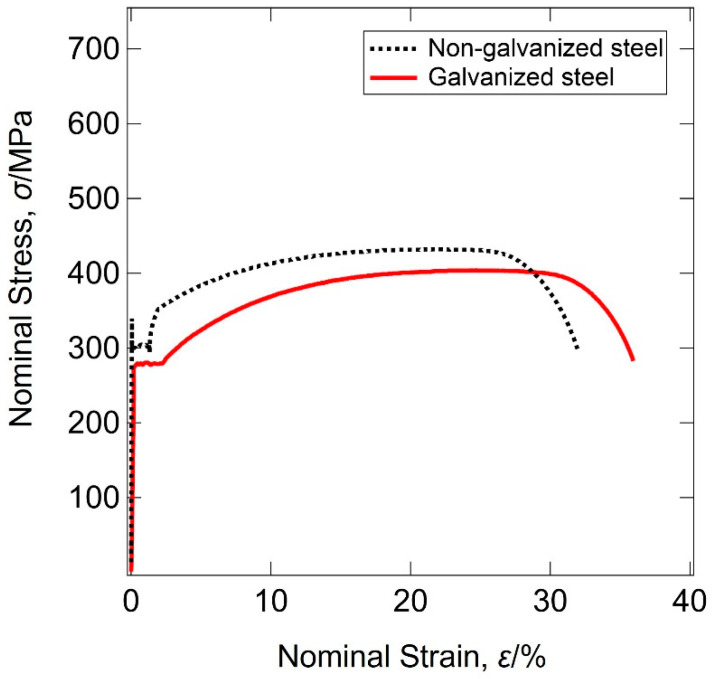
Stress–strain curves for the normalized AISI 1020 steel before and after hot-dip galvanizing.

**Figure 5:**
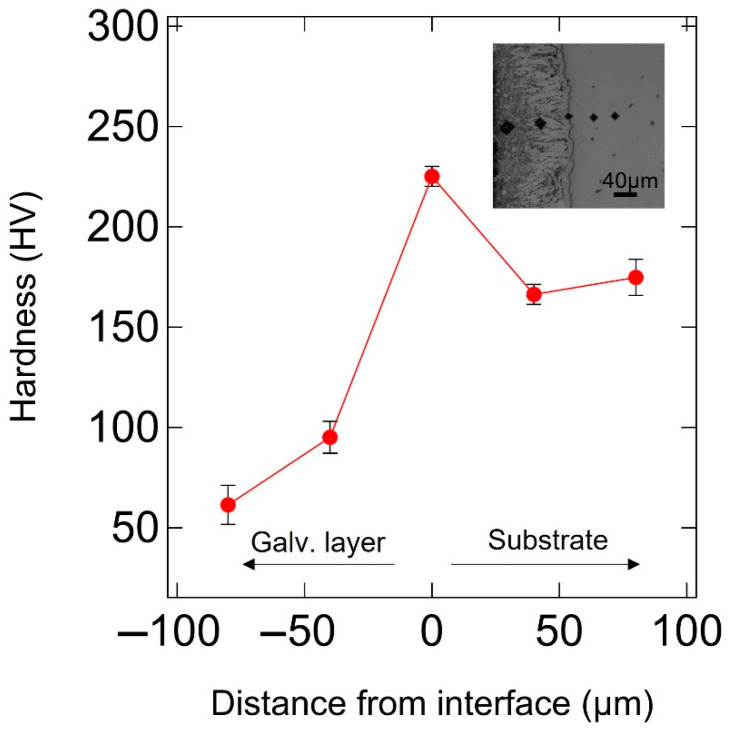
Vickers micro-hardness measurements across the plating layer and substrate interface from the outermost eta phase to the substrate.

The results of tensile tests revealed that the non-galvanized steel had an upper yield strength ($\sigma_{UYS}$) of 340 MPa, a lower yield strength ($\sigma_{LYS}$) of 297 MPa, an ultimate tensile strength ($\sigma_{TS}$) of 432 MPa, and an elongation of 32%. The galvanized steel had a yield strength ($\sigma_{YS}$) of 280 MPa with no lower yield point, an ultimate tensile strength ($\sigma_{TS}$) of 404 MPa, and an elongation of 36% (Figure 4). The hardness test results of the substrates revealed an average hardness value of 175.2 Hv for non-galvanized steel and 168.4 Hv for galvanized steel. The slight difference in tensile properties and Vickers hardness values between non-galvanized and galvanized steel agreed with their microstructures. The Vickers’ hardness values measured across the galvanizing layer from the eta phase to the substrate ranged from 61 to 174 Hv. The Vickers’ hardness of the interface is the largest, and the Vickers’ hardness values of the galvanized layer were less than the substrate hardness, as shown in Figure 5.

### 3.3. Fatigue at Different Stress Ratios

**Figure 6:**
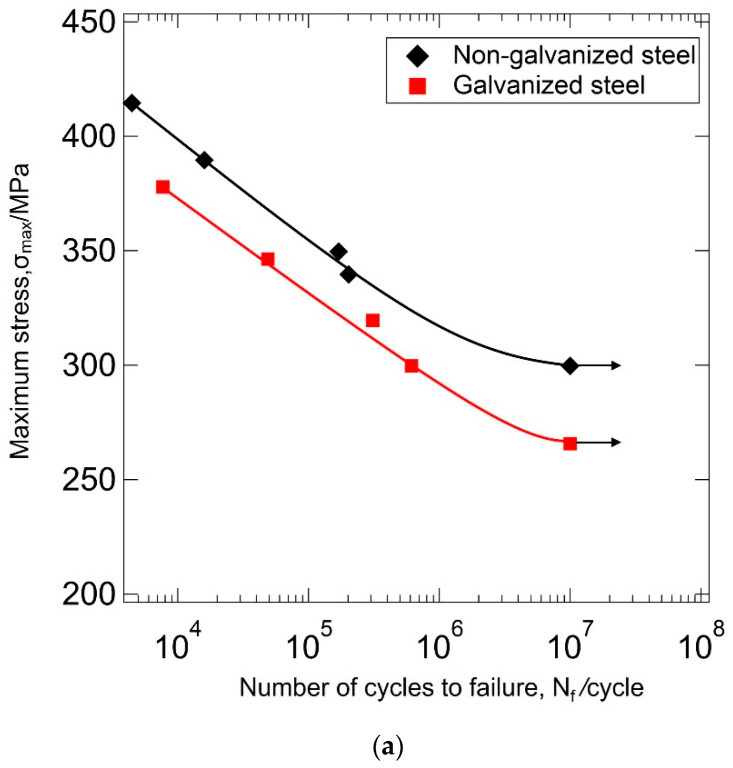

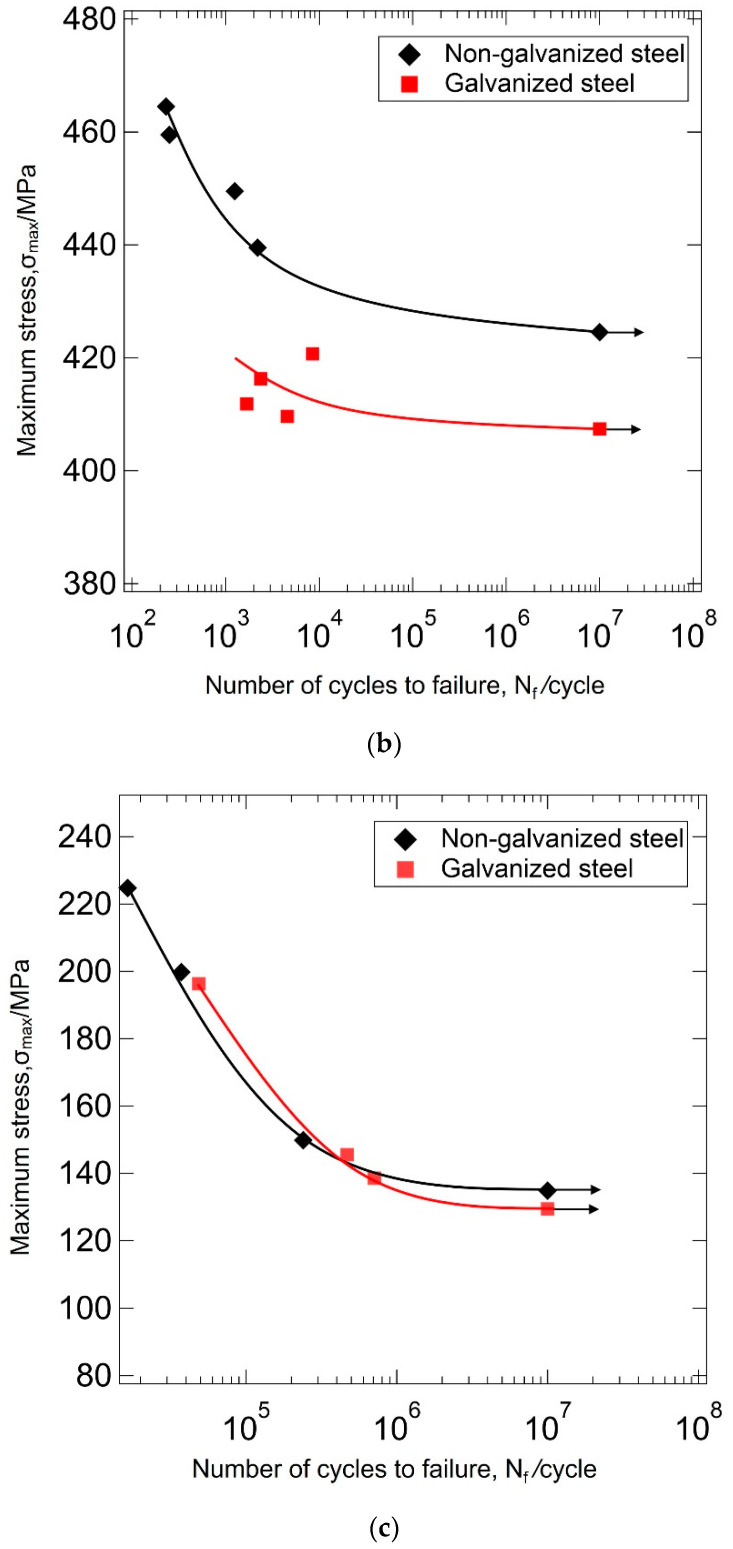
Fatigue strength at *N_f_* = 10^7^ cycles for galvanized and non-galvanized AISI 1020 normalized steels under (**a**) *R* = 0.01, (**b**) *R* = 0.5, and (**c**) *R* = −1.0. Arrows show that the specimen reached 10^7^ cycles, and the test was stopped voluntarily.

Figure 6a shows the stress–life curves at *R* = 0.01. In the high cycle fatigue (10^5^ cycles ≤ *N_f_* ≤ 10^7^ cycles), the number of cycles to failure for the non-galvanized and galvanized steel were 1.60649 × 10^5^ cycles at maximum stress of 350 MPa and 323 MPa, respectively. In the low cycle fatigue (10^3^ cycles ≤ *N_f_* < 10^5^ cycles), the number of cycles to failure for the non-galvanized and galvanized steel were 1.5970 × 10^4^ cycles at maximum stress of 390 MPa and 364 MPa, respectively. Figure 6b shows the stress–life curves at *R* = 0.5. The number of cycles to failure in the high cycle fatigue for the non-galvanized and galvanized steel were 8.10000 × 10^5^ cycles at maximum stress of 426 MPa and 408 MPa, respectively. In the low cycle fatigue, the number of cycles to failure for the non-galvanized and galvanized steel were 2.370 × 10^3^ cycles at maximum stress of 438 MPa and 416 MPa, respectively. The stress–life curves at *R* = 0.01 and 0.5 show a significant difference in the fatigue strength between the galvanized and non-galvanized steels both in the low and high cycle fatigue. At *R* = −1.0, the galvanizing layer seems to have an insignificant effect on the fatigue performance in the low and high cycle fatigue because the number of cycles to failure are somewhat identical, as shown in Figure 6c. It is worth noting that the purpose of this paper is to present the effect of the galvanizing layer on the fatigue strength, and not to ascertain the fatigue limit, since that is affected by the significant scatter that is normally associated with fatigue test experiments. The arrows show that the specimen reached 10^7^ cycles, and the test was voluntarily stopped thereafter.

### 3.4. Fractography at the Different Stress Ratios

**Figure 7:**
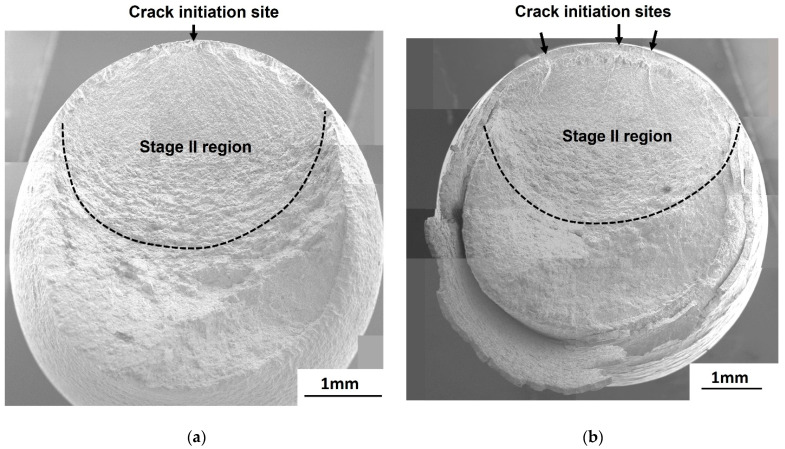
Fatigue fracture surfaces of normalized AISI 1020 steel in the high cycle fatigue at *R* = 0.01: (**a**) non-galvanized, $\sigma_{max}$ = 339.6 MPa and (**b**) galvanized, $\sigma_{max}$ = 319.5 MPa.

**Figure 8:**
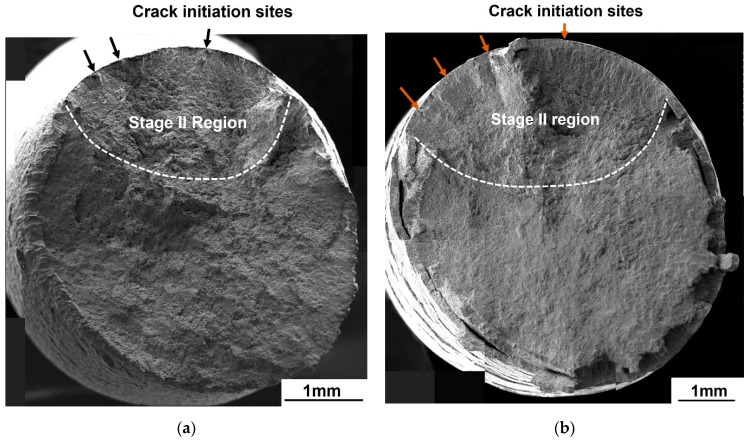
Fatigue fracture surfaces of normalized AISI 1020 steel in the low cycle fatigue at *R* = 0.01: (**a**) non-galvanized, $\sigma_{max}$ = 389.6 MPa and (**b**) galvanized, $\sigma_{max}$ = 346.4 MPa.

At *R* = 0.01, the fracture surfaces for galvanized steel were different from those of non-galvanized steel. In the high cycle fatigue (10^5^ cycles ≤ *N_f_* ≤ 10^7^ cycles), the fracture surface of the non-galvanized steel revealed a flat and single crack initiation site with an oval-shaped crack propagation (stage II) area (Figure 7a), while the galvanized steel exhibited three ratchet marks, which are traces of crack growth from multiple crack initiation sites, also with an oval-shaped crack propagation (stage II) area (Figure 7b). In the low cycle fatigue (10^3^ cycles ≤ *N_f_* < 10^5^ cycles), the galvanized steel exhibited more ratchet marks, signifying an increase in the number of crack initiation sites, and a step was also observed, which indicates the growth and merging of two macrocracks, as shown in Figure 8a. For the non-galvanized steel, ratchet marks with multiple crack initiation sites were observed in the low cycle fatigue (Figure 8b). 

**Figure 9:**
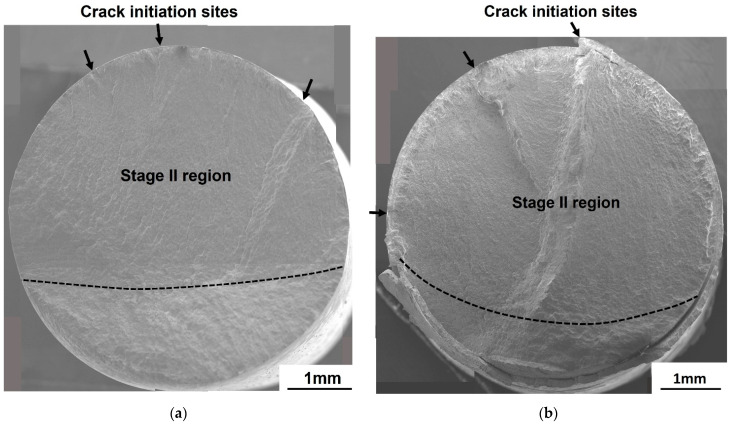
Fatigue fracture surfaces of normalized AISI 1020 steel in the high cycle fatigue at *R* = −1.0: (**a**) non-galvanized, $\sigma_{max}$ = 149.8 MPa and (**b**) galvanized, $\sigma_{max}$ = 138.5 MPa.

**Figure 10:**
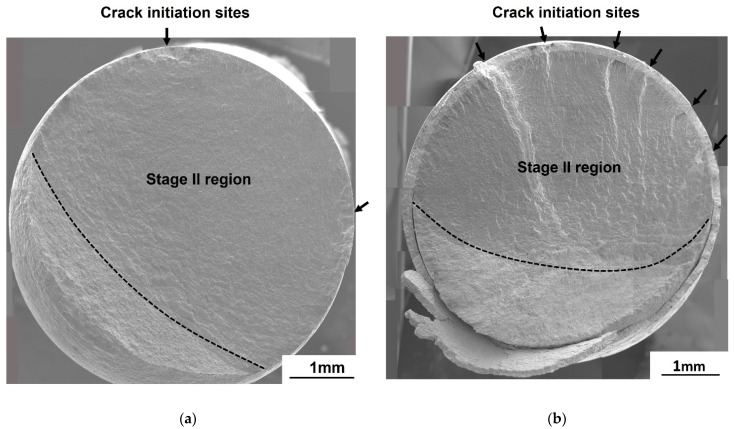
Fatigue fracture surfaces of normalized AISI 1020 steel in the low cycle fatigue at *R* = −1.0: (**a**) non-galvanized, $\sigma_{max}$ = 224.8 MPa and (**b**) galvanized, $\sigma_{max}$ = 196.3 MPa.

At *R* = −1.0, both the galvanized and the non-galvanized steels exhibited multiple crack initiation sites observed from the ratchet marks in the high cycle fatigue (10^5^ cycles ≤ *N_f_* ≤ 10^7^ cycles), as shown in Figure 9. The number of initiation sites seems to be independent of the stress for non-galvanized steel (Figure 10a). However, the number of crack initiation sites increased in the low cycle fatigue (10^3^ cycles ≤ *N_f_* < 10^5^ cycles) for galvanized steel, which was evident from the increase in the ratchet marks (Figure 10b). The fracture surfaces show steps, which result when adjacent cracks merge. The size of the steps is dependent on the stress state, with large steps resulting from low stress while smaller steps result from high stress.

**Figure 11:**
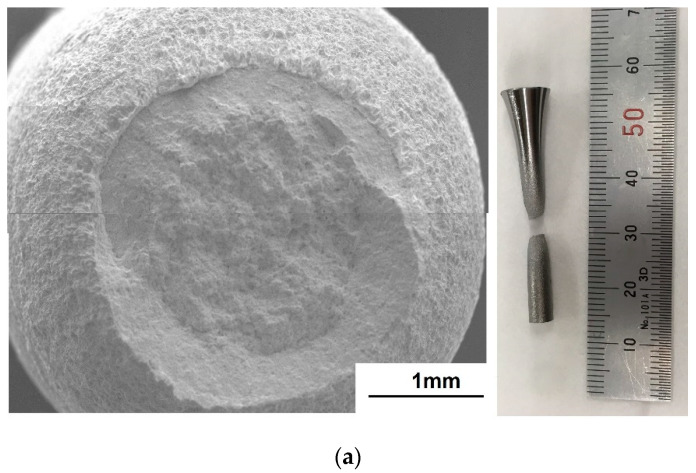

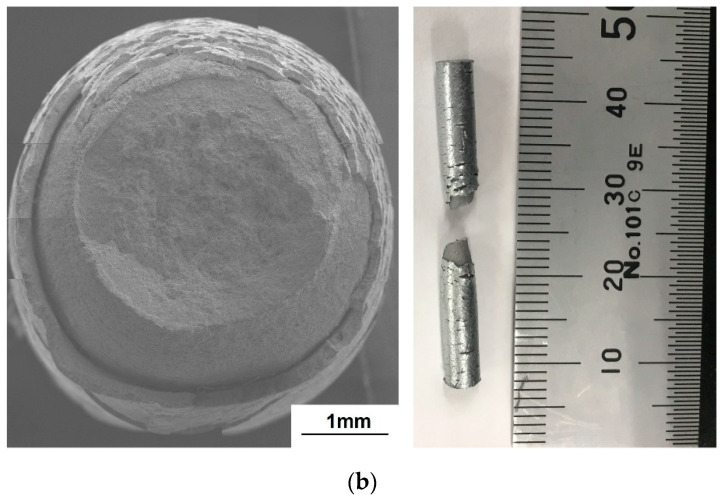
Fracture surfaces and optical photographs from the side near the failure site for normalized AISI 1020 steel at *R* = 0.5: (**a**) non-galvanized, $\sigma_{max}$ = 414.5 MPa and (**b**) galvanized, $\sigma_{max}$ = 377.8 MPa.

At *R* = 0.5, the fracture surfaces of both the non-galvanized and galvanized steels were identical in the low cycle fatigue (Figure 11). All the observed specimens failed in the low cycle fatigue or ran out at 10^7^ cycles. The photographs next to the fracture surfaces reveal an elongation near the failure site for both the non-galvanized and galvanized steels, which appears to be a ductile fracture of both specimens. However, for the galvanized steel, many cracks on the galvanized layer at the sites far from the failure site (non-elongated part) were observed. It suggests that the galvanizing layer may have influenced the fatigue mechanism at *R* = 0.5.

### 3.5. Crack Initiation and Subcracks

**Figure 12:**
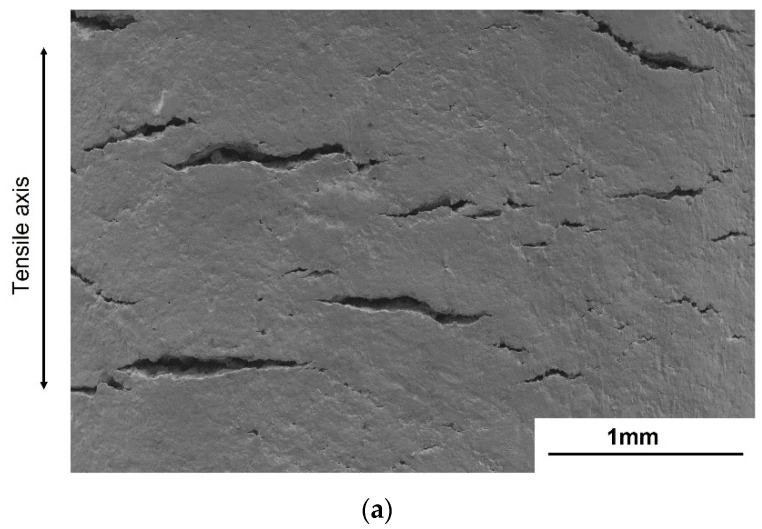

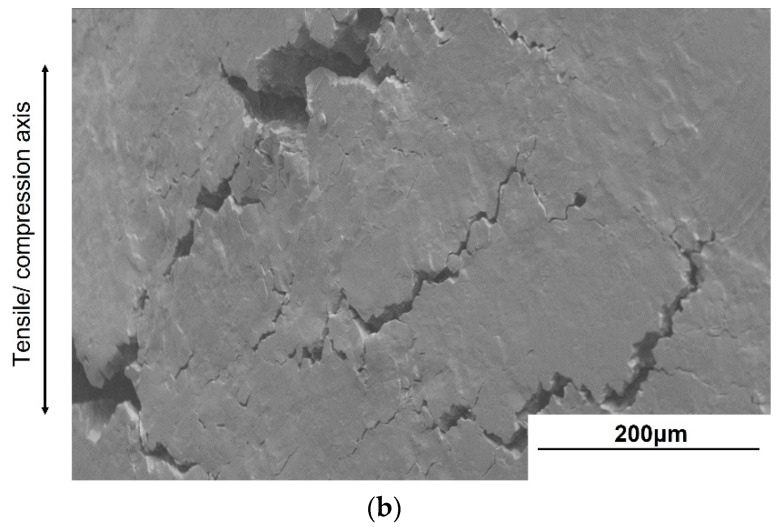
SEM images of the subcracks on the side surface of galvanized steel at (**a**) *R* = 0.01 and (**b**) *R* = −1.0.

**Figure 13:**
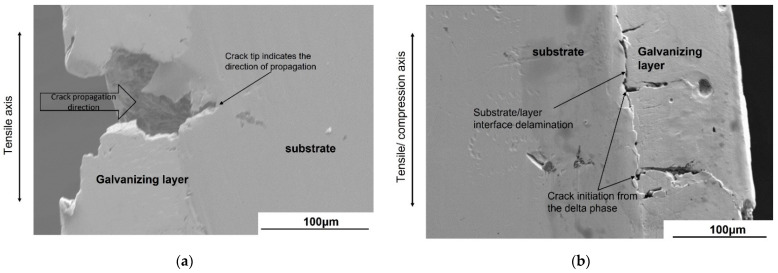
SEM images of the subcracks at the galvanizing layer/substrate interface: (**a**) Subcracks grow from the surface to the substrate at *R* = 0.01, and (**b**) delamination and subcracks originating from the delta phase at *R* = −1.0.

To understand how the stress ratio affects the fatigue strength of galvanized steel, the subcracks on the surface of the fatigued specimens were scrutinized. Careful examination of the exterior surface of the galvanized specimens reveals that the subcracks at *R* = 0.01 were mostly perpendicular to the stress axis, while those at *R* = −1.0 were tilted 45 degrees to the stress axis and exhibited a herringbone pattern. Moreover, the subcracks at *R* = 0.01 and 0.5 were in the range of millimeters, while those at *R* = −1.0 were in the range of micrometers (Figure 11 and Figure 12). Figure 13 compares the subcracks under tension–compression (*R* = −1.0) and tension–tension (*R* = 0.01) loading after sectioning the specimens to study their behavior at the galvanizing layer–substrate interface and to determine their origin. The subcracks at *R* = 0.01 appear to have originated from the surface of the galvanizing layer and propagated into the substrate (Figure 13a). On the other hand, the subcracks originated from the delta phase layer at *R* = −1.0 and propagated to the interface between the galvanizing layer and the substrate or zeta phase layer. Consequently, the fatigued specimen at *R* = −1.0 reveals delamination of the galvanizing layer at the galvanizing layer/substrate interface (Figure 13b).

## 4. Discussion

### 4.1. Effect of the Plating Layer on the Tensile Properties

Hot-dip galvanizing causes a heat effect on steel substrates due to elevated molten zinc bath temperatures and high dislocation density in cold-worked steels. In this study, normalized steel was used to minimize susceptibility to the heat effect as much as possible: the tensile strength of AISI 1020 normalized steel slightly decreased after hot-dip galvanizing, and the hardness test results of the substrates show minor difference between galvanized and non-galvanized steel. In addition, the grain sizes of the galvanized and non-galvanized steel were not so significantly different (Figure 3). The effect of the average grain size on the strength is insignificant from the viewpoint of the Hall–Petch relationship [23]. The upper yield point disappeared, and the discontinuous yielding transformed to continuous yielding, while elongation increased by over 5% (Figure 4). Previous studies reported that the yield and tensile strengths decreased due to the plating layer [24] and the tensile properties of the specimens after removing the plating layer were similar to those before galvanizing [25]. Thus, the decrease in their strength and the increase in elongation were not mainly due to the heat effect on the substrate during galvanizing but were due to the mechanical properties of the plating layer. Generally, plastic deformation begins on the surface before spreading to the bulk of the material. The galvanizing layer is softer than the substrate (Figure 5). The eta phase is the softest of the galvanizing layer [26] and the outermost of all phases of the galvanized steel (Figure 3). Therefore, the initial yielding occurred in the eta phase that is comprised of pure zinc, which exhibits continuous yielding [27], and then, deformation spread to the steel substrate. This agrees with the disappearance of the higher yield point and the appearance of only a lower yield point on the stress–strain curve of galvanized steel (Figure 4).

### 4.2. Effect of Cyclic Deformation Mode on the Fatigue Strength of Galvanized Steel

The role of the cyclic stresses on fatigue strength of unnotched steel specimens at positive stress ratios has been explained sufficiently in terms of crack initiation from the materials surface, and crack propagation rate, *da/dN*, due to the stress intensity factor range at the crack tip [28,29]. Since the applied stress in the low cycle fatigue life is sufficiently large to induce local yielding and subcracks on the surfaces of both the galvanized and non-galvanized steels, the fatigue life can be explained from the Paris’ law related to crack growth in stage II, which means that the effect of the galvanizing layer seems to be insignificant. In fact, there was no significant difference in fatigue strength between the galvanized and non-galvanized steel at *R* = −1.0 in the low cycle fatigue (Figure 6c). This supports the notion that the fatigue life depends on the crack propagation rate of the substrate with or without the galvanizing layer in the low cycle fatigue at *R* = −1.0. However, the fatigue strengths of galvanized steel at *R* = 0.01 and 0.5 were significantly lower than those of non-galvanized steel in the low cycle fatigue (Figure 6a,b). In the previous study, the fatigue strengths of galvanized AISI 1045 cold-worked steel at *R* = 0.01 [30] and a galvanized hot-rolled steel micro-alloyed with titanium and niobium at *R* = 0.05 [31] became significantly lower than those of as-worked steel. It suggests that there is rapid formation of a main crack (growth of subcrack) in the galvanizing layer during the initial cycling of the galvanized steel under tension–tension loading in low cycle fatigue, and as a result, the fatigue strength decreased.

In the high cycle fatigue, it is well known that fatigue life is dependent on the microstructure and surface conditions as they affect damage accumulation, stress concentration, and crack initiation processes on the material’s surface (stage I) [32,33]. In this case, most of the material’s fatigue life is spent on the initiation and growth of microcracks and not on the propagation of the macrocrack. A previous study [30] investigated the effect of the fatigue strength of the cold-worked steel with and without galvanizing, and the results supported that the reduction in fatigue strength was not due to the propagation of the macrocrack but due to the initiation and growth of microcracks. In this study, the fatigue strengths of galvanized normalized steel at *R* = 0.01 and 0.5 decreased in comparison with those of non-galvanized steel, and the results agree with the previous studies [13,16,18,30,31]. On the other hand, the fatigue strength at *R* = −1.0 was the same as that of non-galvanized steel. In addition, the galvanizing layer affects the fatigue strength under the tension–tension fatigue not only in the high cycle fatigue but also in the low cycle fatigue life. It is worth mentioning that detailed surface roughness was not inspected on the test specimens, and cracks can also initiate from surface imperfections, which can influence fatigue results. However, the visual inspections done by SEM on some specimens do not reveal any serious surface roughness of the galvanized steel or any major pre-existing defects when compared to non-galvanized specimen. Therefore, we believe that any reductions in fatigue strength were due mainly to the properties of the galvanizing layer.

### 4.3. Effect of Subcracks Initiated from the Galvanizing Layer on the Microcrack Propagation

Many subcracks on the galvanized layer were observed at all stress ratios (*R* = 0.01, 0.5, and −1.0), as shown in Figure 11 and Figure 12. The subcracks at *R* = 0.01 and 0.5 were mainly perpendicular to the principal stress, while those at *R* = −1.0 tilted to 45 degrees to the principal stress. This suggests that the loading mode that led to the subcracks at *R* = 0.01 and 0.5 was mode I, and that at *R* = −1.0 was mixed modes. The subcracks at *R* = 0.01 initiated from the eta phase of the galvanizing layer and propagated to the steel substrate (Figure 13a). On the other hand, the subcracks at *R* = −1.0 initiated from the delta phase of the galvanizing layer and propagated to the interface between the delta phase and substrate and/or zeta phase layer (Figure 13b). Since the subcracks that initiated in the galvanizing layer propagated to the substrate only under tension–tension loading, the fatigue life decreased at *R* = 0.01 and 0.5. In the bending deformation caused by the compression cycle of the tension–compression loading, the cracks initiated at the substrate/galvanizing layer interface and no crack nucleated in the eta, delta, and zeta phases and propagated to delta and zeta phases [34]. Thus, the crack initiation site and the path of crack propagation depend on the deformation mode.

At *R* = 0.5 in the case of galvanized steel, the fatigue strength in the low cycle fatigue was very close to that in the high cycle fatigue. Since the maximum stress was close to the tensile stress and is superimposed on a mean stress that is also close to the yield stress, ductile fracture was the governing fracture mechanism, and no or very short fatigue crack propagation is expected. The fracture surface of the substrate resembled ductile failure in both the non-galvanized steel and the galvanized one (Figure 11). From their fracture surfaces, it would be plausible that the fatigue strengths of galvanized steel and non-galvanized steel would be similar. However, the fatigue strength of galvanized steel was lower than that of non-galvanized steel (Figure 6b). There were many subcracks formed on the surface of the galvanized steel (Figure 11), which may have induced stress concentration, and the crack propagation rate in the substrate may have become greater. The subcracks may induce stress concentration, and the crack propagation rate in the substrate may increase. More studies are needed to investigate the fatigue process at *R* = 0.5.

## 5. Conclusions

This experimental study was focused on determining the effect of hot-dip galvanizing on the fatigue strength of normalized AISI 1020 steel. Moreover, the study sought to investigate whether the fatigue performance of such steel was dependent on the stress ratio. Finally, a stress range diagram was developed for this steel to address the lack of design recommendations for hot-dip galvanized steels. The following conclusions were drawn:

1. The reduction in fatigue strength by the galvanizing layer was more pronounced in tension–tension fatigue (*R* = 0.01 and 0.5). The reduction in fatigue strength was remarkable in the low cycle fatigue at *R* = 0.5, while it was remarkable in the high cycle fatigue at *R* = 0.01. The fatigue strength was not significantly affected by the galvanizing layer under tension–compression fatigue (*R* = −1.0).
2. The subcracks in tension–tension fatigue initiated from the eta phase of the galvanizing layer and propagated to substrate. The subcracks in tension–compression fatigue initiated from the delta phase of the galvanizing layer, which is at the layer/substrate interface and propagated to zeta and eta phases. Since the propagation of subcracks from the galvanizing layer to the substrate was only evident in tension–tension fatigue, the fatigue strengths in tension–tension fatigue decreased.

## Figures and Tables

**Table 1:** Chemical composition of AISI 1020 steel used in this study (mass %).

C	Si	Mn	P	S	Cu	Ni	Cr	Fe
0.20	0.21	0.35	0.16	0.16	0.01	0.02	0.02	Bal.

**Table 2:** Ferrite grain and pearlite block sizes for normalized AISI 1020 steel before and after galvanizing.

Microstructure	Ferrite Grain Size (µm)	Pearlite Block Size (µm)	Interlamellar Spacing (µm)
Non-galvanized steel	11.4	5.2	0.4
Galvanized steel	12.6	6.1	0.4

## Data Availability

The data presented in this study are available on request from the corresponding author. The data are not publicly available due to intellectual property rights of the collaborating company.
